# Modeling mGluR1 mediated synaptic depression in cerebellar Purkinje cells

**DOI:** 10.1186/1471-2202-15-S1-P110

**Published:** 2014-07-21

**Authors:** Yizhen Su, Huo Lu

**Affiliations:** 1 Doctor of Osteopathic Medicine – GA-Philadelphia College of Osteopathic Medicine, Suwanee, GA 30024, USA; 2 Department of Biomedical Sciences, Philadelphia College of Osteopathic Medicine, Suwanee, GA 30024, USA

## 

We have previously successfully simulated mGluR1 mediated sEPSP based on experimental data. This effect is associated with parallel fiber – Purkinje cell LTD [1-3]. The mGluR1 mediated sEPSP is generated by calcium signaling through the TRPC channel which is crucial in cerebellar LTD induction [4]. Behavior study using mutant mice that lack this type of LTD has shown no motor learning impairment [5]. We hypothesize that cerebellar TRPC mediated synaptic depression shifts the excitatory and inhibitory balance to down regulate ongoing simple-spike activity. To test our hypothesis we modified our previous model of a Purkinje cell to have TRPC channel current signal linked to the AMPA channel conductance through Kinetikit.

**Figure 1:**
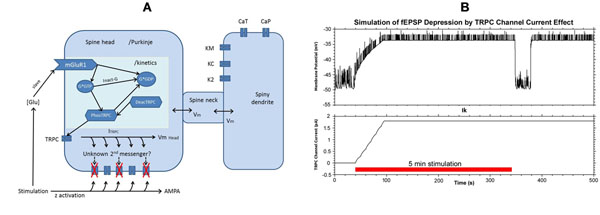
Local model constructed to simulate fEPSP depression triggered by TRPC channel current. **A.** TRPC channel current (Ik) increase due to activation of mGluR1 is linked to AMPA channel conductance (gmax) to simulate the decrease of the channel density. **B.** fEPSPs are evoked randomly at 1 Hz to monitor the amplitude change of the depression effect caused by TRPC current (Ik).

The synaptic depression mediated by TRPC channel current is successfully simulated in the local model. Synchronized train stimulation to the spines in the full model of Purkinje cell were able to cause the cell to fire then followed by a gap in spiking caused by the reduction in the gmax of AMPA channels. Once the TRPC current passed the rising phase, the firing resumed. This model will be used to guide the *in vitro* experiments to study the interaction of TRPC current mediated depression with simple spike activities. Once the second messenger(s) and the delay time of the plastic effects are know, this model can be further used to study the function of cerebellar LTD.
